# Relationship between environmental radiation and radioactivity and childhood thyroid cancer found in Fukushima health management survey

**DOI:** 10.1038/s41598-020-60999-z

**Published:** 2020-03-05

**Authors:** H. Toki, T. Wada, Y. Manabe, S. Hirota, T. Higuchi, I. Tanihata, K. Satoh, M. Bando

**Affiliations:** 1 Research Center for Nuclear Physics, Osaka University, Ibaraki, Osaka 567-0047 Japan; 2 Department of Pure and Applied Physics, Faculty of Engineering Science, Kansai University, Suita, Osaka 564-8680 Japan; 3 Division of Sustainable Energy and Environmental Engineering, Graduate School of Engineering, Osaka University, Suita, Osaka 565-0871 Japan; 4 Institute for Radiation Biology and Medicine, Hiroshima University, Hiroshima, 734-8551 Japan; 5 School of Foreign Service, Georgetown University, Washington, DC. 20057 USA; 6 The Center for Data Science Education and Research, Shiga University, Hikone, 522-8522 Japan; 7 Yukawa Institute for Theoretical Physics, Kyoto University, Kyoto, 606-8502 Japan

**Keywords:** Cancer screening, Risk factors

## Abstract

Environmental radioactive contamination caused by the Fukushima Dai-ichi Nuclear Power Plant accident has aroused great concern regarding a possible increase in the incidence of childhood thyroid cancer. The ultrasound examinations were conducted immediately after the accident as part of the Fukushima Health Management Survey (FHMS), which is divided into the preliminary baseline survey (PBLS) and the full-scale survey (FSS). Some of their outcomes are reported regularly and made available to the public. We have detailed measurements of the air-dose rates and radioactive elements in soil in many places all over the Fukushima prefecture. To study the dose-response relationship, we begin with the assumption that the external and internal doses are correlated with the air-dose rate and the amount of ^131^I in soil, respectively. We then investigate the relationship between these estimated doses and the PBLS and FSS thyroid cancer cases. Our analysis shows that the dose-response curve with the FSS data clearly differs from that with the PBLS data. Finally, we consider the potential mitigating effects of evacuation from highly contaminated areas in both external and internal exposure scenarios.

## Introduction

The earthquake on March 11, 2011 triggered a serious accident at Fukushima Dai-ichi Nuclear Power Plant (NPP). Since then, there has been considerable debate over the possible effects of radiation exposure on human health. Although much is known about immediate damage caused by acute exposure, we still need to make efforts to understand the long-term health risks of exposure to low-dose radiation^1^. It is reported that low-dose radiation could raise the risk of thyroid cancer, especially for young children^1–3^.

A strong association between internal exposure from ^131^I and childhood thyroid cancer became widely known after the Chernobyl accident. It is important to note, however, that epidemiological studies on radiation-induced thyroid diseases predated the Chernobyl accident^4–7^ and continued thereafter^8–10^. These early studies suggested that external medical exposure of the head or neck in childhood raised the risk of thyroid cancer. Much of the data came from the period between the 1920s and the 1960s when it was rather common to apply radiotherapy to some types of diseases in childhood, such as enlarged thymus, tinea capitis and so on. In 1995, Ron *et al*. published the result of an international joint re-analysis of seven existing datasets, including the survivors of the atomic bomb explosions in Hiroshima and Nagasaki^10^. Their result, expressed in terms of the dose-response relationship, has shown that the risk of thyroid cancer due to external exposure was far more serious for children than for adults.

The accident that occurred at the Chernobyl NPP in April 1986 in present-day Ukraine resulted in widespread radioactive contamination^11^. A few years later, the frequent incidence of thyroid cancer was reported among young children from heavily contaminated areas in Ukraine and Belarus^3,12^. A few more years had passed before the international community recognized this serious situation and initiated a systematic survey of childhood thyroid cancer along with dosimetry of radioactive iodine ^131^I focusing on fresh milk as a main route of exposure^13^. The 2000 UNSCEAR report included the estimate that several thousand children in Belarus might have received at least 2 Gy of ^131^I in their thyroid glands^2^. In the 1996 report of the World Health Organization (WHO)^14^, many experts still expressed their doubts about an alleged association between reported increases in thyroid cancer incidence and low-dose radiation exposure, citing as a reason the lack of a definitive study on the dose-response relationship. Then, in 2005, Cardis *et al*.^15^ reported the result of a population-based, case-control study of thyroid cancer in Belarus and Russia, demonstrating a strong dose-response relationship. This finding, among others, finally brought the international scientific community to the conclusion that exposure to ^131^I in childhood is associated with an increased risk of thyroid cancer^16^.

Now the situation in Fukushima is said to be very different from the case of Chernobyl. The estimated radiation doses are much lower than those reported in Hiroshima, Nagasaki, and Chernobyl, namely less than 20 mSv per year in most of the municipalities except for those near the Fukushima NPP^17^. It must be noted that many people evacuated from some of the most contaminated areas shortly after the beginning of the accident. It is also said that, due to the iodine-rich Japanese diet, people in Fukushima may have much lower intakes of ^131^I than those affected by the Chernobyl accident^18^. Nonetheless, it is important to obtain an accurate estimation of possible radiation-related health risks, especially thyroid gland anomaly and thyroid cancer.

The first step toward this end was taken when the prefectural government of Fukushima launched the Fukushima Health Management Survey (FHMS) immediately after the nuclear event. This survey includes, among others, the thyroid ultrasound examinations (TUE) of children at the age of 18 and below who resided in or visited the prefecture at the time of the accident^19,20^. The TUE program is divided into two parts: the preliminary baseline survey (PBLS) and the full-scale survey (FSS). The PBLS is essential because, at the time of the accident, there was no epidemiological data in Japan using ultrasonography. Studies have suggested that the minimum latency period for radiation-induced thyroid cancer is 3-5 years^3,15^. The data from both surveys are regularly updated, and summaries of the data are made available to public through the bulletins of the advisory board for the FHMS committee. Moreover, the Ministry of Environment sponsored a TUE program in three prefectures far from the disaster-stricken areas (Aomori, Yamanashi and Nagasaki: AYN) with the same screening and diagnostic protocols as those used in Fukushima^21,22^. This provides us with control data to be compared with that of the FHMS.

Tsuda *et al*.^23^ and Ohira *et al*.^24^ respectively have made the first attempts at the epidemiological analysis of the PBLS based on its preliminary results, and Suzuki^25^ has published an interim progress report on the TUE program. These studies have drawn markedly different conclusions regarding the relationship between the incidence of childhood thyroid cancer reported in Fukushima and low-dose radiation exposure. These different conclusions originate from the interpretation of the screening effect^26,27^. However, it is important to note that an important task ahead is to elucidate the dose-response relationship as suggested by the history of the scientific controversy over the health effects of the Chernobyl disaster. Without this, we cannot determine whether or not the observed excess risk is attributable to radiation exposure.

Thanks to the nuclear physics community, we have detailed deposition maps of gamma-ray emitting radioactive nuclides in eastern Japan based on extensive soil sampling in addition to air dose measurements conducted shortly after the Fukushima accident. The result of this soil survey was published by Saito, Tanihata and others, with help of over 700 scientists and students mobilized through several organizations^28,29^. These large amounts of radiation datasets, combined with the epidemiological data from the PBLS and the FSS, provide us with a strong quantitative basis to investigate the relationship between radiation doses and the thyroid cancer cases detected in both the PBLS and FSS. Although the TUE program is still underway, we believe that the data obtained so far allow us to make useful observation of such a relationship.

In this paper, we investigate the possible effects of the Fukushima NPP accident on the incidence of childhood thyroid cancer in Fukushima. In Section 2, we describe the screening test of the TUE program conducted in Fukushima immediately after the accident. In Section 3, we summarize the dose distribution based on the soil sampling and air dose survey conducted in Fukushima. We tabulate the air-dose rate and also the amounts of ^134^Cs, ^137^Cs, and ^131^I in soil for each municipality. In Section 4, we proceed to probe the relationship between environmental radioactivity and the thyroid cancer cases observed in the PBLS and the FSS. We examine the reported thyroid cancer incidence against the distribution of air-dose rates and also against the amounts of ^131^I. In Section 5, we study the dose-response relationship in the external and internal radiation exposure scenarios with the information currently available in the literature on 59 municipalities using the Poisson regression method with the standard R software. Section 6 is devoted to the summary of the present study.

## Screening of Childhood Thyroid Cancer

**Figure 1:**
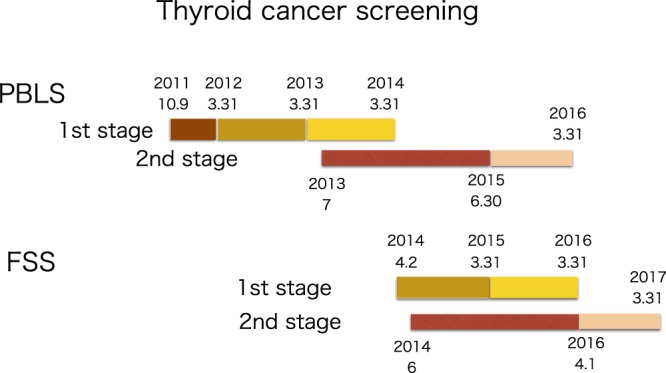
The time schedule of the thyroid cancer screening program. The Fukushima Dai-ichi NPP accident occurred on March 11, 2011. The PBLS is divided into two parts. The primary examination started on October 9, 2011 in areas with the highest air doses and then moved to those with intermediate- and low-level contamination until it covered the entire prefecture by March 31, 2014. The numbers indicate the date in order of year-month-day. In some cases with two numbers, the information on day is missing. The three regions classified for screening are indicated by different colors. The reexamination of those found to have some thyroid anomaly took place during the period from July 2013 to March 31, 2016. The FSS follows a similar two-stage schedule: the primary examination was conducted between April 2, 2014 and March 31, 2016, and the second stage took place during the period from June 2014 to March 31, 2017.

The TUE program in Fukushima began on October 9, 2011 with the aim of screening children at the age of 18 and below who lived in or visited the prefecture at the time of the nuclear reactor accident. Although the participation was voluntary, over 300,000 children signed up for the program. The time schedule of the PBLS and the FSS is shown in Fig. 1.

Each survey proceeded through two stages. First, all participants were screened for any anomaly with the thyroid. Those with nodules and/or cysts larger than a certain size were then subject to further examinations. The primary examinations in the PBLS were carried out between October 9, 2011 and March 31, 2014. To give priority to those presumably at the highest risk, the prefecture was divided into three zones with different air-dose rates, and the survey began with the most contaminated areas and then moved to those with lower doses. The reexaminations of those diagnosed with some thyroid anomaly took place during the period starting from July 2013 until March 31, 2016. For the FSS, the primary examinations began on April 2, 2014 and continued through March 31, 2016, and the reexamination phase starting from June 2014 up to March 31, 2017. To judge whether or not there are any health effects of the nuclear reactor accident, it is important to compare the results of these two surveys with information obtained from other parts of Japan located far away from the path of the radioactive plume arising from the destroyed reactors. We note here that the measurements of air-dose rates to be used for the dose-response relationship in this paper were performed in June and July 2011.

The further survey (third survey, which is called the second FSS) started on May 1, 2016. By the time of December 31, 2018, about 220,000 children participated to the screening program. In this paper, we do not include the data from the third survey.

## Radiation Distribution in Fukushima Prefecture

To investigate the relationship between the amounts of radiation and the thyroid cancer cases in the PBLS and the FSS, we first offer an overview of the radiation data in Fukushima. Shortly after the nuclear reactor accident, a thorough survey was conducted with regard to radioactive materials in soil and air-dose rates at 1,734 sites across Fukushima. The amounts of ^134^Cs, ^137^Cs and ^131^I are provided in the paper of Saito *et al*. and also on the website of the Japan Atomic Energy Commission^28,29^. It should be noted that the amounts of radioactive materials shown in these documents are calibrated to the date of June 14, 2011, taking account of the half-lives of ^131^I (8.02 days), ^134^Cs (2.06 years) and ^137^Cs (30.2 years).

Of course, it would be ideal if we were to have information on the directly measured thyroid doses of all children who underwent the PBLS and FSS in order to discuss the dose-response relationship. A dosimetry study with 62 evacuees conducted shortly after the Fukushima accident has shown that their estimated equivalent thyroid doses were much smaller than the mean thyroid dose in the Chernobyl accident^17^. The UNSCEAR reports also contain information about many similar efforts to estimate the thyroid doses due to the Fukushima accident^30,31^. However, these data are too few and uncertain to be extrapolated for direct comparison to the results of the ultrasound screening program that involved some hundreds of thousands of people from all municipalities. Since a complete dataset of individual doses are currently unavailable and are unlikely to come in the foreseeable future, we proceed with the assumption that the external and the internal doses of the participants in the screening program are correlated with the air dose-rates and the amounts of ^131^I in soil in their original locations at the time of the accident, respectively. We then try to refine our dose estimates by considering the mitigating effects of evacuation and also on the basis of the existing studies with small numbers of samples.

We calculate the air-dose rate and the amounts of key radionuclides for each municipality (village, town, and city) in Fukushima prefecture using the data of the air-dose-rate measurements and the soil studies^28,29^. For this purpose, we take the average of the values listed in the tables on the website of the Japan Atomic Energy Commission^29^ for each municipality (see Tables 1 and 2 in Supplement). These tables show that radiation levels are relatively high in municipalities hosting or near the Fukushima Dai-ichi NPP, and that the levels are very low in those located far west. For example, the air-dose rates in Okuma, Futaba and Namie are 17.79, 17.68 and 14.43 *μ*Sv/h, respectively. In contrast, those in Hinoemata, Shimogo and Nishiaizu are 0.08, 0.08 and 0.09 *μ*Sv/h, respectively. There are several municipalities with large numbers of children who participated in the health survey, for which the mean air-dose rates are low and moderate. Conversely, there are relatively small numbers of participants in the areas with high mean air-dose rates.

We decided first to use the air-dose rate and the amount of ^131^I in soil for comparison with the thyroid cancer cases reported in the PBLS and FSS. The data from the soil study in Tables 1 and 2 in the supplement show that the amounts of ^134^Cs and ^137^Cs are perfectly correlated with each other. On the other hand, the amounts of ^131^I seem to consist of two separate portions, which presumably came from different sources due to the multiplicity of explosions that occurred at the Fukushima Dai-ichi NPP^28^.

To appraise the relative importance of ^131^I in the overall radiological situation in Fukushima, we calculate the integrated absorbed dose of each major radioactive element found in soil in the first year starting from March 15 or March 20, 2011, considering the short half-life (8.02 days) of ^131^I. This comparison is meant only for the external dose. The integrated absorbed dose from ^131^I is about one-third for the March 15 assumption and one-fifth for March 20 assumption as compared to the sum of those from ^134^Cs and ^137^Cs, where the amount of ^137^Cs is 1.2 times larger than that of ^134^Cs. In most municipalities, the contribution of ^131^I is about one-third to one-fifth of that of the Cs isotopes. It must be noted, however, that some municipalities like Iwaki have larger contributions of ^131^I than others. If we were to take the March 15 assumption, the amount of ^131^I is comparable to the radiation dose due to the Cs isotopes in Iwaki. As for the internal dose, we explore the correlation between the thyroid cancer cases and the amounts of ^131^I in municipalities. In fact, the internal doses estimated in the UNSCEAR report are correlated with the amounts of ^131^I in soil^30,31^. We should keep in mind, however, that the internal thyroid dose depends largely on the place that a child visited in the early days of the accident and also on one’s dietary habit following the accident due to the short life time (8.02 days) of ^131^I and its specific exposure pathways, namely inhalation and ingestion.

In addition to the measurements of air-dose rates and radioactivity in soil in Fukushima prefecture performed in June and July 2011, there are airborne radiation measurements using helicopters flying at an altitude of about 150~300 m. The airborne measurements are carried out occasionally and still continue to date^32^. The comparison of the results measured on the ground^28^ against those of airborne measurements shows that radiation levels continued to decline after the soil survey was conducted. We take the data of airborne measurements from June 2011 and compare them with those of the air-dose rates measured on the ground. In most places, the rates reported by the airborne sampling program are similar to those from the soil survey. In some cases, however, difference reaches about a factor of two in both directions.

## Method

### Area distributions of the thyroid cancer cases

With the thyroid cancer and radiation dosimetry information discussed above in hand, we now proceed to examine the relationship between the reported thyroid cancer cases and the estimated doses of radiation. Under the circumstances mentioned in the previous sections, the most comprehensive and reliable information to be used as substitutes for the thyroid doses are the air-dose rates and the amounts of radioactive materials in soil corrected to June 14, 2011. We know the cancer cases for each municipality and the air dose information by taking average over many environmental radiological measurements made within the community. There are, however, many municipalities where the number of cancer cases is zero or small due to the fact that the number of children in the community concerned is small.

**Figure 2:**
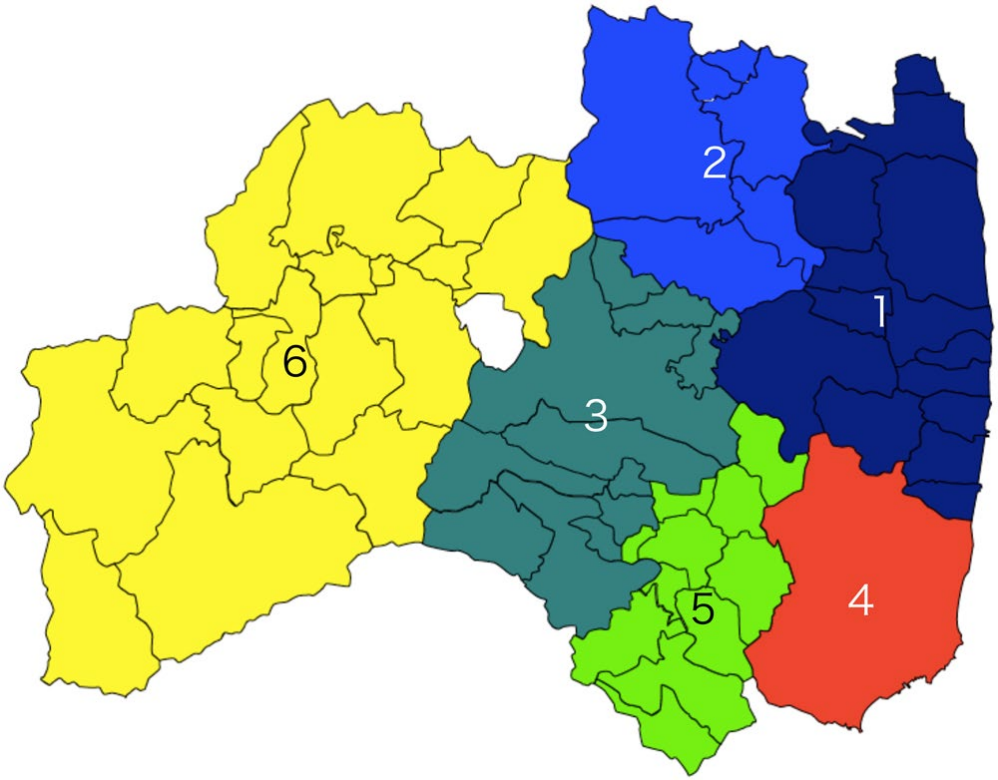
The municipalities of Fukushima prefecture are grouped into six areas differing in radiation levels. In deciding how to divide the prefecture, we also ensure that the child population size in each area is large enough to contain a reasonable number of thyroid cancer cases. These areas serve as a basis for studying the relationship between the incidence of thyroid cancer and the amounts of radiation. Iwaki city is classified as an area of its own given its relatively large child population and amounts of ^131^I. The white spot in this map is Lake Inawashiro. The identification numbers assigned to the six areas are in order of radiation levels.

The numbers of children vary greatly from one township to another, whereas the environmental radiation levels change rather gradually. We therefore group neighboring municipalities of similar radiation levels so that each area has the child population large enough to contain a meaningful number of thyroid cancer cases. Based on the analysis of both populations and radiation distributions, the whole prefecture is divided into six areas as indicated by different colors in Fig. 2. The names of the municipalities in these six areas are listed in Table 1. The six areas are numbered according to environmental radiation levels from the highest to the lowest as shown in the map of Fukushima prefecture (Fig. 2). These levels, based on both air-dose rates and amounts of ^131^I, are listed in Table 2 together with the number of thyroid cancer cases in each of the six areas. To calculate the environmental radiation and radioactivity level in each area, we use Tables 1 and 2 in Supplement and take an average of the air-dose rate and of the radioactivity of ^131^I in soil weighted by the number of children who participated in the FSS survey as shown in Table 2 ^33–35^. The six areas have a few notable characteristics. For example, the air-dose rates vary largely from 0.18 *μ*Sv/h to 4.23 *μ*Sv/h. The number of children in each area is chosen to range between 12,247 and 86,981. The amount of ^131^I is relatively large in Area 4, which corresponds to Iwaki.

**Table 1:** List of the municipalities in six areas, which are shown in Fig. 2.

Area	Municipality
1	Namie, Iitate, Minamisoma, Tamura, Hirono, Naraha, Tomioka, Kawauchi, Okuma, Futaba, Katsurao, Soma, Shinchi
2	Kawamata, Date, Fukushima, Nihonmatsu, Koori, Kunimi
3	Motomiya, Otama, Koriyama, Ten-ei, Shirakawa, Nishigo, Izumizaki, Miharu, Sukagawa, Kagamiishi, Yabuki
4	Iwaki
5	Nakajima, Ishikawa, Yamatsuri, Asakawa, Hirata, Tanagura, Hanawa, Samegawa, Ono, Tamakawa, Furudono
6	Hinoemata, Minamiaizu, Kaneyama, Showa, Mishima, Shimogo, Kitakata, Nishiaizu, Tadami, Inawashiro, Bandai, Kitashiobara, Aizumisato, Aizubange, Yanaizu, Aizuwakamatsu, Yugawa

**Table 2:** The numbers of children who participated in the PBLS and the FSS of the TUE program and the thyroid cancer cases for FSS and for PBLS in the six areas classified on the basis of radiation levels. The air-dose rate and the amount of radioactivity of ^131^I in each area are shown in the 6th and 7th columns. The values in brackets (*n*) show the number of children in each of the six areas who are formally diagnosed with thyroid cancer (malignant or suspected) following the secondary examination. The values $n{\prime}$ are the corrected numbers using Eq. (1).

Area	FSS	Cancer	PBLS	Cancer	Air-dose rate	^131^I
	(Total)	${\boldsymbol{n}}{\prime}$(*n*)	(Total)	${\boldsymbol{n}}{\prime}$(*n*)	[*μ*Sv/h]	[kBq/m^2^]
1	29,471	12.8 (11)	35,344	11.1 (10)	4.23	6.08
2	64,339	22.1 (19)	72,299	22.1 (21)	1.07	0.92
3	86,981	30.8 (25)	96,541	47.6 (44)	0.74	0.37
4	45,265	11.0 (9)	49,430	25.4 (24)	0.34	0.88
5	12,247	2.4 (2)	13,139	4.4 (4)	0.27	0.15
6	32,207	6.7 (5)	33,720	13.1 (12)	0.18	0.45

In Table 2, the cases of thyroid cancer (malignant or suspected) noted in the final report of the PBLS and of the FSS presented in the June 2017 meeting of the Oversight Committee are shown in brackets for each area. These represent the actual cases diagnosed as thyroid cancer in the secondary examination. Since the number of children who actually underwent the examination is smaller, particularly in the FSS, than the number of children who were assigned for the examination, we simply assume that the number of cancer cases is proportional to the number of examinees. This assumption is supported by the fact that the numbers of cancer cases are correlated very well with the numbers of children assigned for the second examination. Thus, we get the corrected cancer incidence ($n{\rm{{\prime} }}$) from the observed cancer case (*n*) in the following way.

1 $$n{\rm{{\prime} }}=n\times \frac{({\rm{n}}{\rm{u}}{\rm{m}}{\rm{b}}{\rm{e}}{\rm{r}}\,{\rm{o}}{\rm{f}}\,{\rm{c}}{\rm{h}}{\rm{i}}{\rm{l}}{\rm{d}}{\rm{r}}{\rm{e}}{\rm{n}}\,{\rm{w}}{\rm{h}}{\rm{o}}\,{\rm{w}}{\rm{e}}{\rm{r}}{\rm{e}}\,{\rm{a}}{\rm{s}}{\rm{s}}{\rm{i}}{\rm{g}}{\rm{n}}{\rm{e}}{\rm{d}}\,{\rm{f}}{\rm{o}}{\rm{r}}\,{\rm{t}}{\rm{h}}{\rm{e}}\,{\rm{s}}{\rm{e}}{\rm{c}}{\rm{o}}{\rm{n}}{\rm{d}}{\rm{a}}{\rm{r}}{\rm{y}}\,{\rm{e}}{\rm{x}}{\rm{a}}{\rm{m}}{\rm{i}}{\rm{n}}{\rm{a}}{\rm{t}}{\rm{i}}{\rm{o}}{\rm{n}})}{({\rm{n}}{\rm{u}}{\rm{m}}{\rm{b}}{\rm{e}}{\rm{r}}\,{\rm{o}}{\rm{f}}\,{\rm{c}}{\rm{h}}{\rm{i}}{\rm{l}}{\rm{d}}{\rm{r}}{\rm{e}}{\rm{n}}\,{\rm{w}}{\rm{h}}{\rm{o}}\,{\rm{f}}{\rm{i}}{\rm{n}}{\rm{i}}{\rm{s}}{\rm{h}}{\rm{e}}{\rm{d}}\,{\rm{t}}{\rm{h}}{\rm{e}}\,{\rm{s}}{\rm{e}}{\rm{c}}{\rm{o}}{\rm{n}}{\rm{d}}{\rm{a}}{\rm{r}}{\rm{y}}\,{\rm{e}}{\rm{x}}{\rm{a}}{\rm{m}}{\rm{i}}{\rm{n}}{\rm{a}}{\rm{t}}{\rm{i}}{\rm{o}}{\rm{n}})}.$$

We have made this correction for the cases observed in the PBLS and the FSS respectively. The results are provided in Table 2 as the number of cancer cases. At first glance, the correction seems to make little difference, as the two values, *n* and $n{\rm{{\prime} }}$, are similar for the latest data from the PBLS and the FSS. However, the two values on the basis of the earlier data at the time of June 2016 had significantly diverged values for area 6: *n* = 1, which provided $n{\rm{{\prime} }}$ = 5.3 using the above relation (1). When the actual number *n* was updated from *n* = 1 to 5, the corrected number becomes $n{\rm{{\prime} }}$ = 6.7 as shown in Table 2. Hence, the corrected number changed only slightly from 5.3 to 6.7, which now roughly corresponds to the actual number. This means that the corrected number is more likely to represent a true value than the actual number which is subject to constant updates as the survey continues. Therefore, we use the corrected number $n{\rm{{\prime} }}$ instead of *n* in the analysis discussed below.

### Relationship between the air-rate doses and ^131^I in soil and the cancer cases reported in the PBLS

In this section, we test the hypothesis that the number of thyroid cancer cases (malignant or suspected) found in the PBLS represents prevalence (natural incidence prior to and during the survey period) because the first screening test mostly completed before a minimum latency period of radiation-induced thyroid cancer (3–5 years) had passed. It is important to note, however, that the determination of the cancer prevalence was made over a long span of time (July 2013 to March 2016) as the survey only gradually covered the entire prefecture.

**Figure 3:**
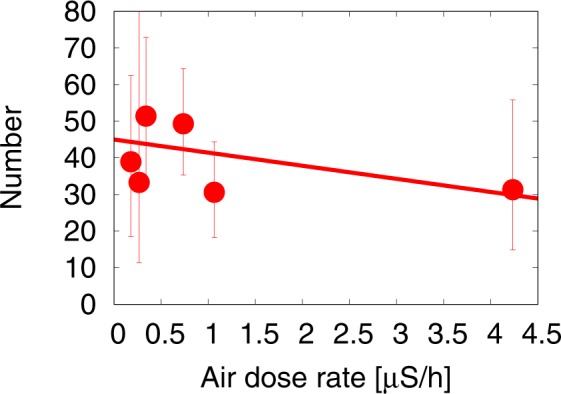
The thyroid cancer case per 10^5^ children *N* in six areas, based on the PBLS data, as a function of the hourly air-dose rate *x* for the six areas. Error bars are shown using the 95% confidence level of the Poisson distribution with the correction factor $n{\rm{{\prime} }}$(1). We include a straight line obtained by the Poisson regression analysis with *N* = *ax* + *b*, which is shown by the solid line: *N* = −3.58*x* + 45.01 with the 95% confidence interval: (−6.81, 1.50) for *a* and (37.97, 52.95) for *b*.

First, we examine the relationship between the air-dose rate and the cancer incidence. Figure 3 using the numbers in Table 2, shows the thyroid cancer case per 10^5^ children *N* as a function of the hourly air-dose rate *x*. We can see a slightly negative correlation between the incidence of cancer and the air-dose rate. We perform a Poisson regression analysis with a straight line *N* = *ax* + *b* with *x* being the air-dose rate [*μ*S/h]. The linear function obtained from the maximum Likelihood distribution is

2 $$N=-3.58\,x+45.01\,,$$

with the 95% confidence interval: (−6.81, 1.50) for *a* and (37.97, 52.95) for *b*, which are obtained using the Likelihood distribution of the Poisson regression analysis. The case of no dose dependence *a* = 0 is included within the 95% confidence interval. If the PBLS represents prevalence, then it is unlikely to depend on the dose rate with statistical significance. This means that the PBLS data does not show a positive effect of external radiation exposure due to the Fukushima accident on the risk of thyroid cancer in the duration of the PBLS survey.

Now, we also examine the relationship between ^131^I in soil and the PBLS data. Unlike the air-dose rate data, the ^131^I data is not available in some municipalities. Therefore, we use the average value of ^131^I in the area that such localities belong as listed in Table 2. We then proceed to perform the Poisson regression analysis of all 59 municipalities with the exponential model (*N* = *e*^*ax*+*b*^), using the generalized linear model (GLM) in the R statistical software. For the further description of this analysis, please see Section 4.5. Table 3 in that sub-section shows that the p-value of *a* is p = 0.93 and the AIC value is 128.4. This indicates that the dependence of the cancer risk observed in the PBLS on ^131^I in soil is essentially zero. This and our earlier analysis of the air-dose rates both support our hypothesis that the cases reported by the PBLS represent the prevalence of thyroid cancer without the influence of radiation exposure due to the reactor accident.

**Table 3:** The results of the Poisson regression analyses with the exponential model (*N* = *e*^*ax*+*b*^) using the GLM command in R for the FSS and PBLS data sets with the air-dose rate and with ^131^I in soil. We provide the coefficients *a* and *b* in the first and second column, the p-value of *a* in the third column, and the AIC value in the fourth column.

Screening	dose	*a*	*b*	p-value of *a*	AIC
FSS	air-dose	0.075	3.37	0.023	104.1
FSS	^131^I	0.022	3.43	0.31	107.0
PBLS	air-dose	0.0081	3.71	0.84	128.4
PBLS	^131^I	0.0019	3.72	0.93	128.4

One may wonder why there is a slight negative tendency against the air-dose rate. It is important to recall that the screening test was conducted in three successive stages, starting in the highest air-dose-rate area and only gradually moving to the lower dose-rate areas as indicated in Fig. 1. The slightly decreasing tendency of the cancer prevalence observed in the PBLS might be due to the time difference in the screening schedule, since the incidence of thyroid cancer increases with age. This effect is mentioned in the paper by Tsuda *et al*.^23^. Regrettably, the available data of the screening test at the time of writing is insufficient to examine this aging effect in further detail.

### Relationship between the air-dose-rates and the cancer cases in the FSS

**Figure 4:**
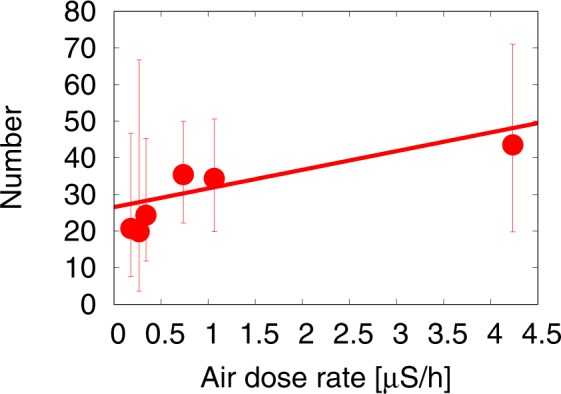
The number of thyroid cancer case per 10^5^ children in six areas, based on the FSS data, as a function of the hourly air-dose rate. These areas are classified according to the formula shown in Fig. 2 and in Table 1. Poisson error bars are included using the method explained in Fig. 3. We include a straight line using all data points with the Poisson regression method: *N* = 5.05*x* + 26.54. We obtain the 95% confidence interval using the Likelihood curve as (0.44, 11.61) for *a* and (19.78, 34.57) for b.

In this subsection, we discuss the relationship between the incidence of thyroid cancer reported in the FSS and the air-dose rate. Fig. 4 shows the case per 10^5^ children *N* in the FSS as a function of the air-dose rate *x*.

We see some increasing tendency in cancer incidence as a function of the air-dose rate. We perform the Poisson regression analysis with *N* = *ax* + *b* linear function, and find the following relationship:

3 $$N=5.05\,x+26.54\,,$$

with the 95% confidence interval using the Likelihood curve as (0.44, 11.61) for *a* and (19.78, 34.57) for *b*. Here, *x* denotes the air-dose rate in [*μ*Sv/h] and *N* the number of cancer cases per 10^5^ children in the FSS. The constant term indicates the cancer risk which does not depend on the dose-rate, with the excess risk provided by the *x* dependent term. We see that the coefficient of the *x* dependent term *a* = 0 is outside of the 95% confidence interval. Fig. 4, however, shows some correlation of the two parameters (*a*, *b*). We calculate the probability of the case of positive *a* value in the likelihood distribution in the *a* − *b* plane and find that the probability is 94.5%.

Now it is important to recall that the air-dose rate is not the actual dose received by children. For the better estimation of the received dose, we need detailed lifestyle information, such as how many hours one spends outdoor in a day. We have some information on the population movement due to the evacuation from the heavily contaminated area, but it is still necessary to obtain more detailed information on the actual doses of individual children^36^. Equally important and uncertain is a mechanism in which cumulative radiation doses change year by year. We address these issues further in Section 5.

### Relationship between ^131^I and cancer cases for the FSS

Finally we consider an alternative scenario in which the main cause of thyroid cancer is not external radiation but rather the intake of ^131^I in the thyroid gland. The amount of ^131^I is particularly large in Iwaki compared with the air-dose rate. This discrepancy underlines the importance of examining the dose-response relationship observed in the FSS with a focus on internal exposure. We note here that the air doses include negligible contributions of ^131^I at the time of measurements in June 2011.

**Figure 5:**
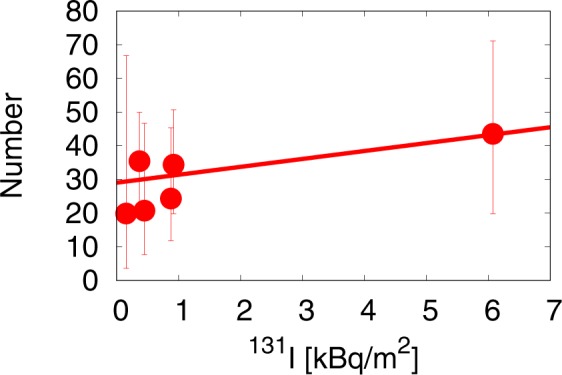
The thyroid cancer case per 10^5^ children observed in the FSS as a function of the amount of radioactivity from ^131^I in six areas. We show a straight line obtained by a Poisson regression analysis: *N* = 2.35*x* + 29.03 with the 95% confidence interval (−0.78, 7.27) for *a* and (22.23, 37.10) for b.

The results of our analysis are shown in Fig. 5, where the thyroid cancer cases reported in the FSS are plotted as a function of the amount of radioactivity from ^131^I. In this instance, we see a positive correlation between the two quantities. We perform a Poisson regression analysis in the same manner as in the case of the air-dose rate, we obtain

4 $$N=2.35\,x+29.03\,,$$

with the 95% confidence interval (−0.78, 7.27) for *a* and (22.23, 37.10) for *b*. The dose dependence is small, and the cancer incidence is dominated by the constant term. The slope is 2.35 but the slope of zero is included in the 95% confidence interval for *a*. It is important to take full account of this result because it has been generally believed that, like in Chernobyl, the intake of ^131^I would be chiefly responsible for the potential excess risk of thyroid cancer in Fukushima. The failure to detect a statistically significant correlation between thyroid cancer and ^131^I in our analysis may be due to the fact that Japanese people tend to take a large amount of stable iodine from seaweed, a diet pattern which is known to reduce the intake of ^131^I significantly. However, it is still important to make better estimates of thyroid doses for individuals, which depend largely on the places of children during and immediately after the Fukushima accident due to the short lifetime of ^131^I.

### Statistical analysis based on the information of 59 municipalities

A question remains as to whether or not our analysis of the relationship between the levels of environmental radiation and radioactivity and the incidence of thyroid cancer depends on the way in which municipalities are grouped into larger areas. Tsuda *et al*.^23^ have divided the prefecture into nine areas, while Ohira *et al*. have designated three areas^24^, and Suzuki five^25^. It is straightforward to calculate environmental radiation and radioactivity levels and cancer cases using Tables 1 and 2 in Supplement as well as the lists provided in the PBLS and the FSS. The results are essentially the same. However, the grouping of municipalities into several areas may add more uncertainty to the statistical analysis.

Hence, we analyze the dose-response relationship from a totally different point of view. These raw data sets contain large error bars for each municipality. Therefore, we use the generalized linear model (GLM) in the R statistical software^37^ to perform the Poisson regression analysis of all 59 municipalities. We take into account the fact that some children assigned for the secondary examination did not get the examination, which amounts to 7 % for the PBLS and 18 % for the FSS. This fact is considered in the six-area analysis by using $n{\rm{{\prime} }}$ instead of *n* as shown in Table 2. In the actual calculation, we simply reduce the number of participants by multiplying $n/n{\rm{{\prime} }}$ to the number of participants while keeping the value of cancer cases integer for the GLM analysis. As for the amount of ^131^I in soil, there are municipalities where no data are available, in which we use the average value of ^131^I in the area they belong as listed in Table 2.

**Figure 6:**
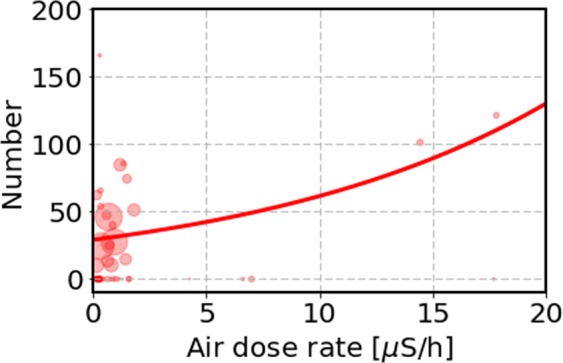
The thyroid cancer case per 10^5^ children observed in the FSS as a function of the amount of air-dose rate in all 59 municipalities. The areas of the circles are proportional to the number of participants in each municipality to indicate the importance of the data points with larger circles. We show an exponential function by the solid curve obtained by a Poisson regression analysis: *N* = *e*^0.075*x*+3.37^ as shown in Table 3.

We show the results of the GLM analysis in Table 3 on the number *N* of cancer cases per 10^5^ children. The first row of the table concerns the FSS with the exponential model, which is the standard model of statistical analysis. The p-value of *a* is p = 0.023 and the Akaike Information Criterion (AIC) value is AIC = 104.1. This indicates that the correlation between the air-dose rate and incidence of the cancer is statistically significant. In order to see the results of the GLM analysis, we show in Fig. 6 the number *N* against the air-dose rate. Since it is difficult to show the Poisson errors for each number in Fig. 6, we vary the areas of each number by the number of participants in each municipality.

We perform a similar Poisson regression analysis for the cancer cases as a function of the amount of ^131^I in soil for the FSS. The results are shown in the second row of Table 3. The p-value of *a* is p = 0.31. This indicates that the ^131^I dependence of the cancer risk is not statistically significant. The AIC value is 107.0, suggesting that the cancer case of the FSS is less likely to be correlated with ^131^I in soil than the air-dose rate, where the AIC value is 104.1.

We perform the Poisson regression analyses with the air-dose rate and the amount of ^131^I for the PBLS. The results are shown in the third and fourth rows of Table 3. The p-value of *a* for the air-dose rate is p = 0.84 and that for ^131^I in soil is p = 0.93. The AIC values are 128.4 for both cases. This indicates that the radiation dependence of the cancer risk for the PBLS is essentially zero.

## External and Internal Doses and Cancer Cases in 59 Municipalities

As we have seen, the incidence of cancer reported in the FSS is correlated with the air-dose rate, while their correlation with the amount of ^131^I in soil is weak. In this section, we try to include more information on external and internal doses obtained from actual radiological measurements of individuals and also from calculations of possible received doses using information regarding the movement of individuals.

Many children evacuated from highly contaminated areas through various places in the Fukushima prefecture within the first several days of the reactor accident to lower dose areas. In fact, the mobile phone analyses were made before and after the Fukushima accident, and it was found that most of the people within 20km from the Fukushima power plant had been indeed evacuated^36^. This fact should affect the incidence of radiation-induced thyroid cancer in the highest air-dose rate area, where the cumulative doses for children should be reduced by the evacuation. The up-to-date information on the actual external and internal doses of children is published in 2017 by Ishikawa^38^. We study this and other publications to obtain information on the thyroid doses for our analysis.

First, we discuss the case of external dosage^39,40^. Ishikawa *et al*. estimate individual external doses by using digitized behavior data from individual questionnaires and a computer program that includes daily dose-rate maps drawn after the accident^40^. This analysis provides the external doses received by those in several municipalities. The authors divide Fukushima into seven areas and provide dose distributions and average doses using the data obtained from 421,394 persons. There are strong correlations between their 7 areas and our 6 areas. More specifically, Area 6 in our grouping is divided to Aizu and Minami-Aizu areas in their grouping. The results are tabulated in Table 4 for average doses in these seven areas in Fukushima. These average doses are calculated for four-month doses (March 11 to July 11, 2011). Two things stand out in this table. The external doses calculated by Ishikawa *et al*. are rather low in all seven areas. If we simply multiply 4 months (2880 hours) by the air-dose rates in the second row, the results are about 3 times larger than the values in the first row except for the Soso area. This factor 3 is mentioned in the literature as due to the effect that people stay indoor for some time in a day^40^. As for the Soso area, Ishikawa et al’s estimate, 0.8 mSv, is much smaller than the amount obtained from the air-dose rate, 14.3 mSv. The ratio is about 18 (=6 × 3). The reason is due largely to the evacuation of people from the highly contaminated area (a factor of approximately 6) and the lifestyle factor of 3.

**Table 4:** Average external doses in mSv for four months in seven areas listed in the first row, reported by Ishikawa *et al*.^40^. For comparison, we list the air-dose rates in *μ*Sv/h in these seven areas in the second row, calculated from Tables 1 and 2 in Supplement.

Area	Kenpoku	Kenchu	Kennan	Aizu	Minami-Aizu	Soso	Iwaki
External dose [mSv]	1.4	1.0	0.6	0.2	0.1	0.8	0.3
Air-dose rate [*μ*Sv/h]	1.10	0.66	0.53	0.22	0.11	4.98	0.34

**Figure 7:**
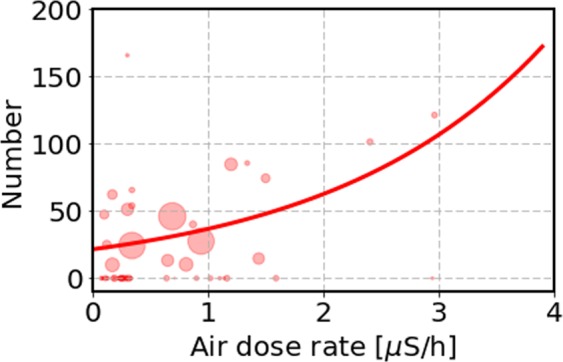
The thyroid cancer case per 10^5^ children observed in the FSS as a function of the amount of air dose rate in all 59 municipalities. Here, we reduced the air-dose rate by a factor 6 for the Area 1 in Table 2. We show an exponential function by the solid curve obtained by a Poisson regression analysis: *N* = *e*^0.533*x*+3.07^ as shown in Table 5.

As a first step to take into account the evacuation effect, we simply multiply the air-dose rate in the highly contaminated area, indicated as Area 1 in Table 2, by 1/6 and keep the air-dose rates unchanged for the other areas. We then make the Poisson regression analysis for all 59 municipalities. The results are shown in Table 5 in the first row, and in Fig. 7. The agreement to the cancer incidence data is improved as indicated by AIC = 102.1 and the p-value is 0.009. It is further important to mention that the number *a* is now 7 times larger than the same value for the direct use of the air-dose rate as shown in the first row in Table 3. This increase of *a* is caused by the data points in Area 1, whose air-dose rates are shifted to smaller side by a factor 6 as can be seen in Fig. 7. This result indicates that the correlation regarding the FSS is larger in the modified case (*a* = 0.533) than the unmodified one. Now, we take the grouping of seven areas for the study of dose-response relationship. The results are shown in the second row in Table 5. We obtain a similar value for *a* = 0.502 as the 59 municipality analysis, and the p-value indicates that the agreement is plausible.

**Table 5:** The results of the Poisson regression analyses with the exponential model (*N* = *e*^*ax*+*b*^) using the GLM command in R for the FSS data set with the external and internal doses. We provide the coefficients *a* and *b* in the first and second column, the p-value of *a* in the third column, and the AIC value in the fourth column. The AIC value in the second row is small due to the fact that we use 7 areas in this analysis.

dose	*a*	*b*	p-value of *a*	AIC
air-dose (modified)	0.533	3.07	0.009	102.1
7 areas with external dose	0.502	3.08	0.12	32.7
UNSCEAR10	0.009	3.24	0.53	107.6
^131^I (modified)	0.109	3.39	0.45	107.4

We now turn our attention to the case of internal dose^38,41–43^. An effort has been made to provide the thyroid doses due to internal exposure, which are tabulated for municipalities in Fukushima in the UNSCEAR report^30,31^. The total internal thyroid doses consist of two pathways, namely inhalation and ingestion. In the UNSCEAR report, the estimated internal thyroid doses due to inhalation vary in 59 municipalities, where the smallest value is 0.03 mSv in Hinoemata and the largest value is 17.76 mSv in Iwaki as one year internal dose for 10-year-old children^31^. On the other hand, the internal thyroid doses due to ingestion are uniform in all municipalities, and the value given in the literature is 15.24 mSv. These internal thyroid doses are much larger than the external doses shown in Table 4. There are other efforts made to improve the estimates of the inhalation doses using the direct measurements of the thyroid doses, whole-body-counter measurements, and estimation using SPEEDI data^38,41–43^. A detailed study of the inhalation doses has been performed for many municipalities, and the upper bound values are not inconsistent from the UNSCEAR average values^42^.

We perform the Poisson regression analysis of the incidence of thyroid cancer observed in the FSS by using the UNSCEAR values of the thyroid doses for 10-year-old children and the results are shown in the third row indicated as UNSCEAR10 in Table 5. The p-value is 0.53 and the AIC value is 107.6, which are slightly worse than AIC = 107.0 in the case of ^131^I in soil shown in Table 3. Since the internal inhalation dose has a strong correlation with ^131^I in soil except for the highly contaminated area, we proceed to make a simple modification by multiplying the ^131^I values by 1/6 in Area 1. The results are shown in the fourth row in Table 5. The agreement is indicated by the value AIC = 107.4, which is close to the AIC value of the UNSCEAR case.

Since the positive correlation of the FSS cancer cases with the internal dose with large contributions from ^131^I is weak and is not statistically significant, we infer that the internal dose is not likely to be the cause of the FSS cancer cases. On the other hand, the FSS cancer cases are correlated largely with the external dose, even after considering the effect of evacuation from the highly contaminated area. However, as listed in Table 4, the estimated external doses are much smaller than the estimated internal doses.

How should we understand this seeming anomaly regarding the dose-response relationship? One possibility might be psychological stress. A study with mice has shown that a mere change in the size of the cage might be enough to increase the risk of cancer^44^. A recent report based on the survey of evacuees in Fukushima several years after the accident has also suggested a possible correlation between the degree of psychological stress and evacuation, the latter associated with the higher degree of radioactive contamination. The average score of the mental test (IESR) is 25.9 for those from the “difficult-to-return” zone, 22.9 for those from the zone where the evacuation order is set to be lifted soon, and 19.8 for those from the zone where the order has been lifted^45^. Those with more than 25 are typically considered to have PTSD. Mental stress, however, is only one of many possible explanations. There are several other reasons mentioned in the literature. One of them might be the obesity effect measured by the BMI, which is influenced by the NPP accident^46,47^. It is also possible that the uncertainty regarding internal exposure to ^131^I might have obscured the true relationship between the thyroid doses and the FSS data. It is therefore important to conduct further investigation into all possible causes, including the effects of radiation exposure, of the observed increase in the incidence of thyroid cancer reported in the FSS as a function of the air-dose rate.

## Conclusion

We studied the thyroid cancer cases in Fukushima after the accident of the Fukushima Dai-ichi NPP in March, 2011. There were two thyroid gland surveys – the preliminary baseline survey (PBLS) and the full-scale survey (FSS) – both using ultrasonography to screen children at the age of 18 and below who were present in Fukushima at the time of the accident. We investigated the relationship between the levels of environmental radiation and radioactivity and the cancer cases based on the data from the PBLS and the FSS on the basis of the information from the soil surveys and air dose-rate measurements.

We conducted two studies on the dose-response relationship. In the first study, we used the air-dose rates and the amounts of ^131^I in soil, which are obtained by direct measurements in many places in Fukushima. In the second study, we used various existing estimates of external and internal doses received by individuals. We also took account of the mitigating effects of evacuation on the radiation exposure of people from highly contaminated areas. The results of the first study are summarized as follows. We found a negative correlation between the thyroid cancer cases in the PBLS and the air-dose rate as well as the amount of ^131^I. We concluded that radiation exposure is unlikely to be responsible for the thyroid cancer detected in the PBLS. As for the FSS, we found a positive correlation between the incidence of thyroid cancer and the air-dose rate. This correlation is statistically significant. These results suggest that the dose-response curve based on the FSS data shows a clearly different structure from that of the PBLS data as long as external radiation exposure is concerned. As for ^131^I, the correlation between its amount in soil and the incidence of thyroid cancer reported in the FSS is very weak. Even the GLM analysis with both the air-dose rate and ^131^I shows that the effect of ^131^I in soil is small.

In the second study, we considered various estimates of external and internal doses from the existing studies. A major determining factor in dose estimates is the evacuation of people from highly contaminated areas. The Poisson regression analysis with the use of the estimated external doses showed a positive dose-response relationship. As for the internal dose, the use of the UNSCEAR values in the Poisson regression analysis showed that the correlation between the internal dose and the incidence of thyroid cancer observed in the FSS is small. These findings are quite puzzling, since the estimated internal doses are much larger than the estimated external doses.

We found a positive correlation between the thyroid cancer cases reported in the FSS and the air-dose rates, with the association stronger with external exposure than with internal one. It is important to continue the study of the dose-response relationship as more data are available. In particular, the extensive dose data collected at 1,734 sites across Fukushima will help us consider the radiation distribution in the further study of the dose-response relationship. We should emphasize here that we ought to perform the study of the internal exposure of individuals and study a relationship between the thyroid doses and the cancer cases. Especially important are quantitative studies of ingestion that contribute to the internal doses. Although it is essential to further refine our understanding of the dose-response relationship, we hope the present analysis provides the basic information on the effect of radiation on the thyroid cancer. We mention here that a similar study on the dose-response relationship between the air-dose rates and the thyroid cancer cases was published recently by Yamamoto *et al*.^48^.

## Supplementary information

Dataset 1.
