# Supplementary material for: Relationship between environmental radiation and radioactivity and childhood thyroid cancer found in Fukushima health management survey
Source: Sci Rep. 2020 Mar 5;10:4074. doi: 10.1038/s41598-020-60999-z (PMC7058088; doi:10.1038/s41598-020-60999-z)
Supplement: Supplementary file 1 — Dataset 1. [file 41598_2020_60999_MOESM1_ESM.pdf]

Supplement of "Relationship between environmental radiation and radioactivity and childhood thyroid cancer found in Fukushima health management survey"

H. Toki[a1], T. Wada[b], Y. Manabe[c], S. Hirota[d], T. Higuchi[e], I. Tanihata[a], K. Satoh[f] and M. Bando[a,g]

[a] *Research Center for Nuclear Physics, Osaka University, Ibaraki, Osaka 567-0047, Japan*
[b] *Department of Pure and Applied Physics, Faculty of Engineering Science, Kansai University, Suita, Osaka 564-8680, Japan*
[c] *Division of Sustainable Energy and Environmental Engineering, Graduate School of Engineering, Osaka University, Suita, Osaka 565-0871, Japan*
[d] *Institute for Radiation Biology and Medicine, Hiroshima University, Hiroshima 734-8551, Japan*
[e] *School of Foreign Service, Georgetown University, Washington DC., 20057, USA*
[f] *The Center for Data Science Education and Research, Shiga University, Hikone 522-8522, Japan*
[g] *Yukawa Institute for Theoretical Physics, Kyoto University, Kyoto 606-8502, Japan*

Abstract

This is a supplemental material of a paper entitled above. We include here tables showing the levels of environmental radiation and radioactivity in all municipalities in Fukushima prefecture.

The tables below show the levels of environmental radiation and radioactivity in all municipalities (cities, towns, and villages) in Fukushima prefecture. Saito, Tanihata *et al.* [*Journal of Environmental Radioactivity* **139**, 308-319 (2015)] collected these data by sampling soil throughout Fukushima and also by measuring the amount of ^{131}I in most parts of the prefecture during the period between June and July 2011. The air-dose rates were measured by dosimetry counters at 1 m high from the ground. The data obtained by this method are in close agreement with the airborne measurements.

[1] toki@rcnp.osaka-u.ac.jp

Table 1: The amounts of radiation recorded in Fukushima prefecture (Part 1). The air-dose rate is measured at 1m high from the ground and shown in units of μSv/h for the period from June 6 to July 8, 2011. The amounts of ^{131}I, ^{134}Cs and ^{137}Cs are obtained from the soil analysis and expresses in units of kBq/m^2 by the time of June 14, 2011, considering that the half-life of ^{131}I is 8.02 days, ^{134}Cs 2.06 years, and ^{137}Cs 30.2 years. The amount of ^{131}I was not measured in some municipalities, which is indicated by – in the slot of ^{131}I.

No.	Municipality	Air dose rate [μSv/h]	^{131}I [kBq/m^2]	^{134}Cs [kBq/m^2]	^{137}Cs [kBq/m^2]
1	Kawamata	1.59	0.931	159	175
2	Namie	14.43	7.13	1680	1860
3	Iitate	6.61	2.18	637	701
4	Minamisoma	1.81	0.820	162	181
5	Date	1.20	0.957	149	158
6	Tamura	0.60	0.502	55.0	58.8
7	Hirono	0.79	1.590	74.7	79.9
8	Naraha	1.58	5.130	113	128
9	Tomioka	6.99	32.0	854	955
10	Kawauchi	1.28	0.408	137	151
11	Okuma	17.79	32.0	2490	2740
12	Futaba	17.68	31.0	2310	2630
13	Katsurao	4.26	1.790	625	714
14	Fukushima	0.94	0.977	146	160
15	Nihonmatsu	1.44	0.628	132	148
16	Motomiya	1.50	0.305	167	183
17	Otama	1.02	0.510	111	122
18	Koriyama	0.69	0.383	91.3	99.5
19	Koori	1.34	0.970	150	168
20	Kunimi	0.90	0.483	116	125
21	Ten-ei	1.14	0.255	110	126
22	Shirakawa	0.65	0.255	63.7	68.1
23	Nishigo	0.87	0.095	80.9	91.8
24	Izumizaki	0.23	0.477	47.2	51.3
25	Miharu	0.64	0.524	75.1	80.9
26	Iwaki	0.34	0.876	26.4	28.7
27	Sukagawa	0.81	0.480	84.8	92.9
28	Soma	0.73	0.230	69.2	77.8
29	Kagamiishi	0.34	0.235	45.9	48.3
30	Shinchi	0.45	0.437	58.2	63.2

Table 2: The amounts of radiation recorded in Fukushima prefecture (Part 2).

No.	Municipality	Air dose rate [μSv/h]	^{131}I [kBq/m^2]	^{134}Cs [kBq/m^2]	^{137}Cs [kBq/m^2]
31	Nakajima	0.30	-	22.9	25.4
32	Yabuki	0.31	-	24.5	27.0
33	Ishikawa	0.25	-	14.1	15.7
34	Yamatsuri	0.12	-	6.08	6.53
35	Asakawa	0.24	-	21.4	24.4
36	Hirata	0.30	0.350	19.2	20.0
37	Tanagura	0.34	0.165	41.4	44.2
38	Hanawa	0.18	-	12.3	13.5
39	Samegawa	0.23	0.0905	20.0	21.5
40	Ono	0.27	0.105	21.0	23.0
41	Tamakawa	0.29	-	16.0	17.6
42	Furudono	0.30	0.0325	21.9	24.2
43	Hinoemata	0.08	-	1.45	1.75
44	Minamiaizu	0.11	-	3.51	3.92
45	Kaneyama	0.11	-	5.58	6.70
46	Showa	0.15	-	17.5	19.0
47	Mishima	0.19	-	12.0	14.0
48	Shimogo	0.08	-	3.47	3.80
49	Kitakata	0.17	0.350	14.2	14.9
50	Nishiaizu	0.09	-	4.23	5.40
51	Tadami	0.13	0.370	4.25	4.73
52	Inawashiro	0.28	-	28.4	30.8
53	Bandai	0.22	-	18.5	20.5
54	Kitashiobara	0.33	-	40.8	46.0
55	Aizumisato	0.19	-	11.5	12.5
56	Aizubange	0.25	-	36.5	40.5
57	Yanaizu	0.15	-	7.43	8.00
58	Aizuwakamatsu	0.17	0.490	18.1	19.7
59	Yugawa	0.30	-	35.0	38.0
